# A QTL of eggplant shapes the rhizosphere bacterial community, co-responsible for resistance to bacterial wilt

**DOI:** 10.1093/hr/uhad272

**Published:** 2023-12-19

**Authors:** Chao Gong, Zhenshuo Wang, Zhiliang Li, Baojuan Sun, Wenlong Luo, Shanwei Luo, Shuting Chen, Peiting Mai, Zhenxing Li, Ye Li, Yikui Wang, Tao Li

**Affiliations:** Guangdong Academy of Agricultural Sciences, Guangdong Key Laboratory for New Technology Research of Vegetables, Vegetable Research Institute, Guangzhou, 510640, China; Department of Plant Pathology, MOA Key Lab of Pest Monitoring and Green Management, College of Plant Protection, China Agricultural University, Beijing 100193, China; Guangdong Academy of Agricultural Sciences, Guangdong Key Laboratory for New Technology Research of Vegetables, Vegetable Research Institute, Guangzhou, 510640, China; Guangdong Academy of Agricultural Sciences, Guangdong Key Laboratory for New Technology Research of Vegetables, Vegetable Research Institute, Guangzhou, 510640, China; Guangdong Academy of Agricultural Sciences, Guangdong Key Laboratory for New Technology Research of Vegetables, Vegetable Research Institute, Guangzhou, 510640, China; Guangdong Academy of Agricultural Sciences, Guangdong Key Laboratory for New Technology Research of Vegetables, Vegetable Research Institute, Guangzhou, 510640, China; Guangdong Academy of Agricultural Sciences, Guangdong Key Laboratory for New Technology Research of Vegetables, Vegetable Research Institute, Guangzhou, 510640, China; Guangdong Academy of Agricultural Sciences, Guangdong Key Laboratory for New Technology Research of Vegetables, Vegetable Research Institute, Guangzhou, 510640, China; Guangdong Academy of Agricultural Sciences, Guangdong Key Laboratory for New Technology Research of Vegetables, Vegetable Research Institute, Guangzhou, 510640, China; Harbin Academy of Agricultural Sciences, Harbin, Heilongjiang, 150029, China; Institute of Vegetable, Guangxi Academy of Agricultural Sciences, Nanning, Guangxi, 530007, China; Guangdong Academy of Agricultural Sciences, Guangdong Key Laboratory for New Technology Research of Vegetables, Vegetable Research Institute, Guangzhou, 510640, China

## Abstract

Resistant crop cultivars can recruit beneficial rhizobacteria to resist disease. However, whether this recruitment is regulated by quantitative trait loci (QTL) is unclear. The role of QTL in recruiting specific bacteria against bacterial wilt (BW) is an important question of practical significance to disease management. Here, to identify QTL controlling BW resistance, Super-BSA was performed in F_2_ plants derived from resistant eggplant cultivar R06112 × susceptible cultivar S55193. The QTL was narrowed down through BC_1_F_1_-BC_3_F_1_ individuals by wilting symptoms and KASP markers. Rhizosphere bacterial composition of R06112, S55193, and resistant individuals EB158 (with the QTL) and susceptible individuals EB327 (without QTL) from BC_2_F_1_ generation were assessed by Illumina sequencing-based analysis, and the activation of plant immunity by the bacterial isolates was analyzed. Evidence showed that BW-resistant is controlled by one QTL located at the 270 kb region on chromosome 10, namely *EBWR10*, and *nsLTPs* as candidate genes confirmed by RNA-Seq. *EBWR10* has a significant effect on rhizobacteria composition and significantly recruits *Bacillus*. pp. A SynCom of three isolated *Bacillus*. pp trains significantly reduced the disease incidence, changed activities of CAT, PPO, and PAL and concentration of NO, H_2_O_2_, and O_2_^−^, activated SA and JA signaling-dependent ISR, and displayed immune activation against *Ralstonia solanacearum* in eggplant. Our findings demonstrate for the first time that the QTL can recruit beneficial rhizobacteria, which jointly promote the suppression of BW. This method charts a path to develop the QTL in resistant cultivar-driven probiotics to ameliorate plant diseases.

## Introduction

Bacterial wilt (BW) caused by *Ralstonia solanacearum* infects about 250 plant species, especially plants from the Solanaceae family, which is considered a major problem in humid tropical and subtropical regions worldwide [1, 2]. To date, various managements have been explored for the control of BW, including breeding resistant cultivars, chemical and biological controls, and soil management, but the control of this disease at a desired level through a sustainable and eco-friendly way is still awaited [2–4]. Breeding-resistant cultivars are still the most logical, economical, and environmental solution for suppressing BW epidemics [2, 3]. Plant resistance to BW is very complex and controlled by QTLs. Genetic analysis using Arabidopsis accession Landsberg *erecta* (L*er*) × Col-0 recombinant inbred lines suggested that *R. solanacearum* resistance is controlled by three loci, namely *QRS1*, *QRS2*, and *QRS3* [5]. Several major QTLs on chromosomes 6, 7, and 10 in *Solanum lycopersicum var. cerasiforme* cultivar L285 [6, 7] and chromosomes 3, 6, 8, 10, and 11 in *S. lycopersicum* cultivar ‘Hawaii 7996’ associated with BW resistance were identified [8–11]. Previous studies have suggested that the resistance of eggplant to BW is monogenic, while studies reported it as polygenic [12]. A major gene, *ERs1* conferring resistance to *R. solanacearum* was identified by *Solanum melongena* MM738 (susceptible) × AG91–25 (resistant) recombinant inbred lines located in chromosome 9 and named *EBWR9*. Two other QTLs were identified on chromosomes 2 and 5, named *EBWR2* and *EBWR5*, respectively [13–15]. Although the main emphasis of BW research is on breeding resistant cultivars has been a challenge for many years [2]. Due to the breeding, cultivars resistant to BW have been restrained by polygenic inheritance, and sometimes the connection between resistance with horticultural undesirable traits related to the wild species (linkage drag) [7, 16]. There is still a need for cultivars with stable resistance, and the genetics of bacterial wilt resistance is still unclear.

Rhizosphere microecological balance may contribute to determining the resistance to BW [17]. Some bacterial taxa have been designated as plant growth-promoting rhizobacteria for BW management [18, 19]. The composition and function of root microbiomes are highly dependent on the plant genotypes and phenotypic traits and the environment in which they live [20, 21]. Plants select microorganisms to colonize in their rhizosphere, and this process is heritable across plant cultivars [22, 23]. The research of Poudel *et al.* [24] highlighted the influence of different rootstock varieties on the composition of the bacteria community in the tomato rhizosphere which proves the potential for selecting bacterial taxa by plant genotype. Studies have shown the influence of genotype on plant-associated microbiome composition in crop varieties and their wild relatives, and gene-specific mutations [21, 25]. Hence, the identification of genes related to specific plant-associated microbial recruitment is the critical step to developing new methods of crop breeding that are able to recruit beneficial microorganisms that support crop health [21], yet the implication of BW resistance genes on rhizosphere microbiome function has been relatively unexplored.

BW of eggplant (*S. melongena* L.) is a major economically destructive disease, causing the yield reduced from 11.67% to 96.67% and even recorded up to 100% loss in humid and congenial climatic conditions [2, 15, 26]. There are few resources for resistance to BW in eggplants available in nature [12]. The eggplant-resistant cultivar R06112 with good commercial properties and a high yield and wide adaptation was used in this study, which could be introgressed into commercial cultivars. The mechanism of BW resistance in eggplant remains scant, restricting resistance breeding and disease management. Therefore, this study aimed to (i) locate the BW resistance locus of eggplant R06112; (ii) decipher the rhizosphere bacterial communities driven by the QTL; (iii) isolate trains significantly recruited by the QTL with strong biocontrol potentials against *R. solanacearum* from rhizosphere soil; and (iv) evaluate BW resistance mechanisms of SynCom composed of isolated trains against *R. solanacearum*.

## Results

### BW resistance inheritance in the eggplant R06112

Chi-squared (χ^2^) analysis was carried out to test the phenotypic data for goodness-of-fit to Mendelian segregation ratios. Two clear-cut BW-response phenotypes, asymptomatic or death were counted in the F_2_ population during 21 dpi. Among 320 F_2_ plants, 94 showed BW susceptibility, and 226 showed BW resistance, with a 3:1 segregation between resistant and susceptible plants (χ^2^ = 3.27, P = 0.07). All F_1_ plants showed BW resistance. The segregating population BC_1_P_s_ and BC_1_P_r_ populations with 1:1 and 1:0 segregation ratios between resistant and susceptible plants (Table S2, see online supplementary material). The results suggested that resistance to *R. solanacearum* in R06112 was controlled by a single dominant gene.

### A monogenic resistance in R06112 with one QTL

**Figure 1:**
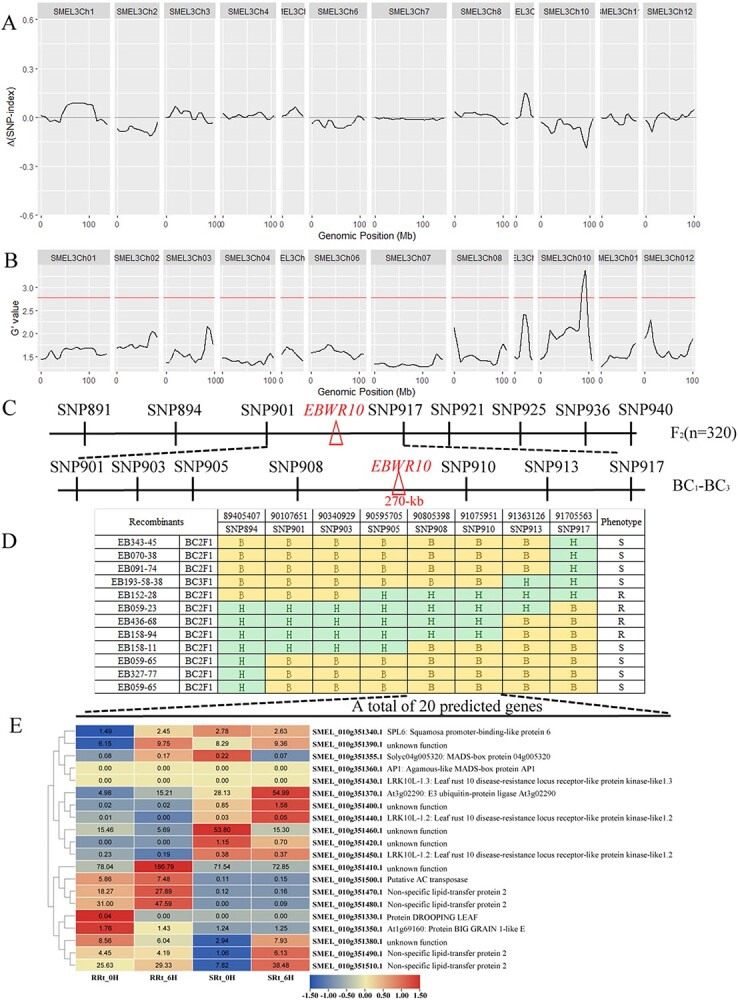
Identification of genetic region(s) in R06112 associated with resistance to BW. **A** and **B**: bulked segregant analysis (BSA) (**A**, Δ (SNP index) of the S- and R-pools; **B**, G’Value of the R- and S-pools. The SMEL3Ch01–12 indicates the 12 eggplant chromosomes. The red line represents the association threshold). **C**–**D**: Further fine mapping with BC_1_F_1_-BC_3_F_1_ plants by KASP markers and BW resistance phenotype delimited *EBWR10* locus into 270 kb genomic region including 20 genes. **E**: The expression levels of genes predicted in the QTL region in R06112 and S51193 by RNA-Seq analysis. RRt-6 h, RRt-0 h: Root of resistant cultivar R06112 infected with *Ralstonia solanacearum* for 6 h, and without *R. solanacearum* infection, respectively; SRt-6 h, SRt-0 h: Root of susceptible cultivar S55193 infected with *R. solanacearum* for 6 h, and without *R. solanacearum* infection, respectively. The numbers represent different expression levels of the genes.

We obtained 60.49 Gb of clean data from the parents and 56.33 Gb from the two mixed pools, with high quality (89.65% > Q30 > 88.70%) and stable GC content (36.51% > GC > 35.79%). The average sequencing depths for two F_2_ pools and parents were 23× and 22×, respectively. In total, 1 712 421 SNPs were identified and after filtering, 201 707 polymorphic SNPs between the resistant and the susceptible DNA bulks were used for association analysis to identify the resistance-related candidate regions in eggplant. A 4.96 Mb region spanning 89.162 and 94.122 Mb on chromosome 10 was identified as the target region associated with BW resistance (Fig. 1A and B). Through the identification of BC_1_F_1_-BC_3_F_1_ individuals by KASP markers and disease resistance in the field, the position of BW resistance QTL was located between SNP908 and SNP910, a 270 kb region, which was designated *EBWR10* (Fig. 1C and D). Twenty genes were annotated in the region, including four non-specific lipid-transfer protein (nsLTPs) genes. The RNA-Seq analysis indicated that the expression levels of two *nsLTPs* genes were significantly upregulated in R06112 by *R. solanacearum* inoculation (Fig. 1E), which was identified as the most likely candidate gene which confers BW resistance in eggplant R06112. Silencing *nsLTP* reduced eggplant R06112 resistance to BW by Virus-induced gene silencing (VIGS) analysis (Fig. S2, see online supplementary material).

### Effect of *EBWR10* on community composition of rhizosphere bacteria

The bacterial microbiota associated with R06112, S51193, and the BC_2_P_s_ individual plants with *EBWR10* (EB158–94) and without *EBWR10* (EB327–77) were evaluated. A total of 1 543 275 high-quality sequences with an average of 64 303 reads in each sample were obtained by Illumina high-throughput sequencing, which was binned (N97% identity) into 9747 operational taxonomic units (OTUs).

**Figure 2:**
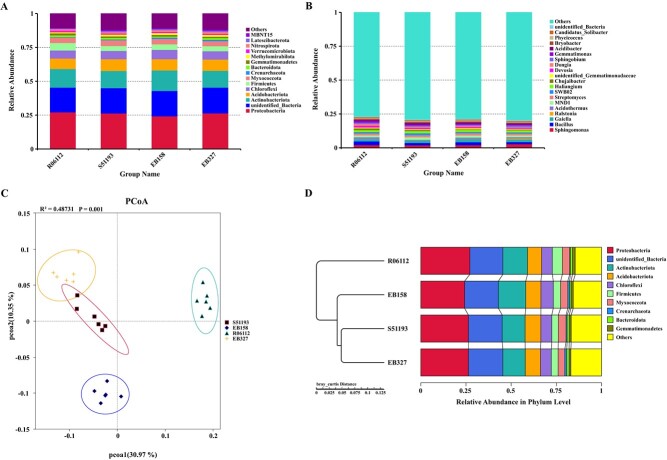
The relative abundance of the most dominant bacterial phyla (**A**) and genera (**B**) in the eggplant rhizosphere. **C**: PCoA of eggplant rhizosphere bacterial community of R06112, S51193, EB158, and EB327, respectively. **D**: Clustering analysis of Bray–Curtis similarity coefficients for bacterial communities based on OTU abundance.

Proteobacteria (24.3–27.1%), Actinobacteriota (12.4–156.1%), Acidobacteriota (7.9–8.7%), Chloroflexi (5.9–16.8%), Firmicutes (3.5–5.5%), Myxococcota (3.7–4.2%), Crenarchaeota (0.4–1.1%), Gemmatimonadetes (0.98–1.5%), Methylomirabilota (0.83–1.4%), and Bacteroidota (0.77–1.3%) were identified as the dominant bacterial phyla existing in all samples (Fig. 2A). *Sphingomonas* (2.0–2.8%), *Bacillus* (1.6–2.7%), *Gaiella* (1.7–2.3%), *Ralstonia* (0.1–0.8%), and *Acidothermus* (0.5–1.2%) were the dominant bacterial genera in all samples (Fig. 2B). The dominant orders in all samples are shown in Fig. S3 (see online supplementary material).

PCoA analysis suggested that the bacterial community structure of R06112 rhizosphere soil was different from EB158, EB327, and S51193. The bacterial community of EB158 tended to separate from EB327 and S51193. Permutational Multivariate Analysis of Variance (PERMANOVA) showed that the bacterial community structure was significantly different among these sample groups (Fig. 2C). The rhizosphere soils clustered into two groups based on bacterial community composition by UPGMA tree, suggesting that the bacterial community of EB158 and EB327 was more similar to the S51193 (Fig. 2D). Shannon and Chao1 of the rhizospheric bacterial community were significantly higher in EB158, and the Shannon index was significantly higher in EB327 than R06112 and S51193 (Table S3, see online supplementary material).

**Figure 3:**
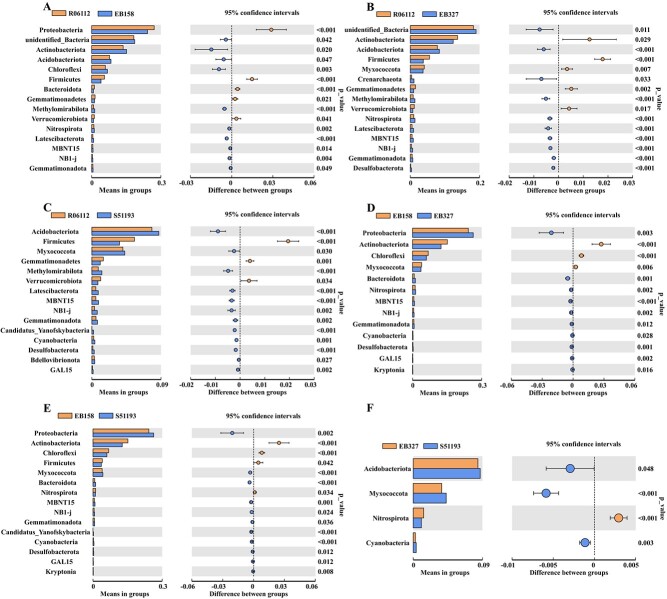
Bacterial phyla whose relative abundance is significantly different between R06112 and EB158 (**A**), R06112 and EB327 (**B**), R06112 and S51193 (**C**), EB158 and EB327 (**D**), EB158 and S51193 (**E**), EB327 and S51193 (**F**) plots.

The relative abundances of Firmicutes and Gemmatimonadetes in the rhizosphere soil of R06112 were significantly higher than that in S51193 and EB327 (*P* < 0.01). The relative abundances of Actinobacteriota and Chloroflexi in the rhizosphere soil of the resistant progeny EB158 were significantly higher than that in susceptible parent S51193 and susceptible progeny EB327 (*P* < 0.01) (Fig. 3). The relative abundance of bacterial orders with significantly different levels is shown in Fig. S4 (see online supplementary material).

**Figure 4:**
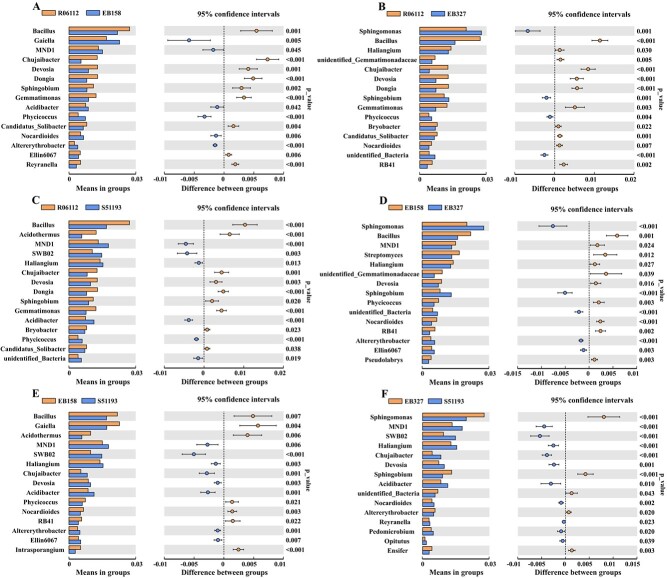
Bacterial genera whose relative abundance is significantly different between R06112 and EB158 (**A**), R06112 and EB327 (**B**), R06112 and S51193 (**C**), EB158 and EB327 (**D**), EB158 and S51193 (**E**), EB327 and S51193 (**F**) plots.

The abundance of *Bacillus* was significantly higher in resistant parent R06112 (with *EBWR10*) and resistant progeny EB158 (with *EBWR10*) than in susceptible progeny EB327 (without *EBWR10*) and susceptible parent S51193 (without *EBWR10*) (Fig. 4). Relative abundances of *Bacillus* were 1.61-fold and 1.29-fold higher in the rhizosphere soil of R06112 and EB158 than in S51193, respectively. The relative abundances of *Bacillus* in the rhizosphere soil of EB327 were similar to that in S51193 (Fig. 4).

### Isolation and identification of antagonistic **B*acillus* strains against *R. solanacearum*

Among 273 isolated strains, three showed strong antagonism against *R. solanacearum* GMI1000 on LB medium (Fig. S5, see online supplementary material). Phylogenetic trees indicate that PR-1 clusters with *Bacillus velezensis*, PR-3 clusters with *Bacillus cereus*, and PR-7 clusters with *B. velezensis* (Figs S6–S8, see online supplementary material). These isolates were named *B. velezensis* PR-1, *B. cereus* PR-3, and *B. velezensis* PR-7.

### Activation of plant immunity by a synthetic community (SynCom)

**Figure 5:**
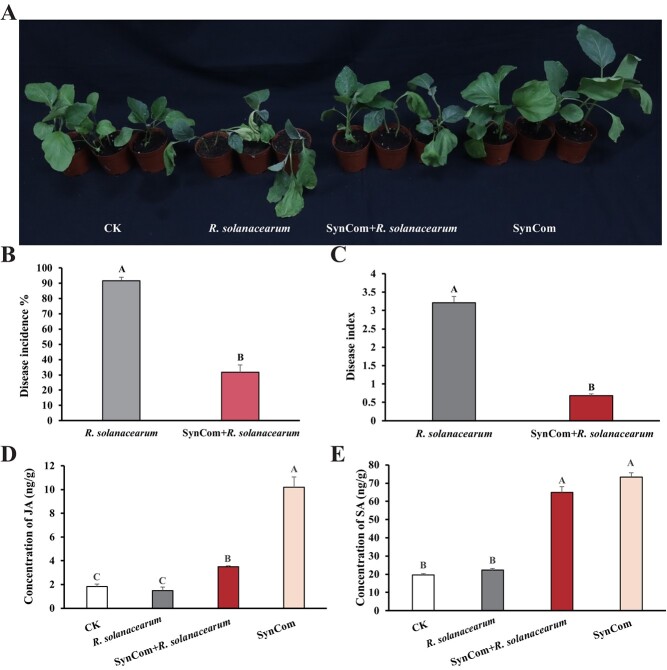
Disease symptoms (**A**), disease incidence (**B**), disease index (**C**), and the concentration of JA (**D**) and SA (**E**) of SynCom on eggplant plants infected with *Ralstonia solanacearum* at 48 hpi. The standard deviation for three independent replicates is represented by error bars. Significant differences between treatments are represented by different letters (*P* < 0.01). SynCom mixture of three *Bacillus* spp. strains.

The disease incidence and disease index of eggplant inoculation with *R. solanacearum* were significantly reduced at 7 dpi by SynCom treated (Fig. 5A–C). In addition, SynCom could promote eggplant growth to a certain extent (Fig. 5A).

To evaluate the physiological effects and functional mechanisms of SynCom on BW resistance, we compared changes in activity of phenylalanine ammonialyase (PAL), catalase (CAT), and polyphenol oxidase (PPO), superoxide (O_2_^−^), nitric oxide (NO), and hydrogen peroxide (H_2_O_2_) content, and phytohormones content including salicylic acid (SA) and jasmonate (JA). Compared to the control, PAL activities were significantly increased in SynCom and *R. solanacearum* treatments at 48 hpi. CAT activities were significantly inhibited with SynCom inducted. PPO activities were increased in SynCom and *R. solanacearum* treatments but decreased in SynCom plus *R. solanacearum* treatment (Fig. S9, see online supplementary material). Compared to *R. solanacearum* treatment, the NO, H_2_O_2_, and O_2_^−^ contents were significantly reduced by SynCom inoculation. The O_2_^−^ level in treatment groups increased significantly compared with the control (Fig. S10, see online supplementary material).

**Figure 6:**
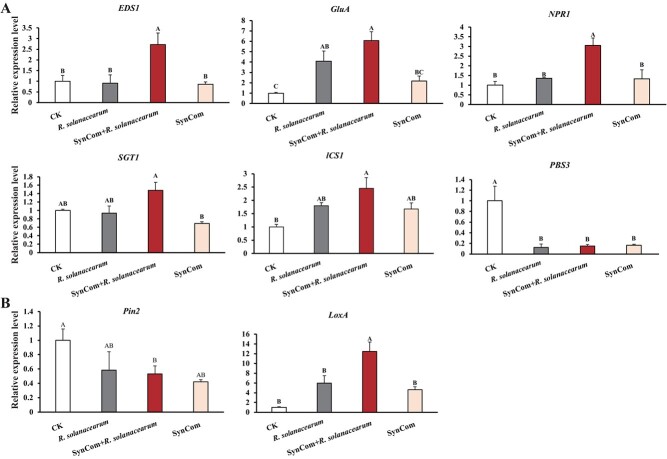
The expression levels of SA (**A**) and JA (**B**) defense signaling marker genes in eggplants infected with *Ralstonia solanacearum* or/and SynCom at 48 hpi by qRT-PCR analysis. The standard deviation for three independent replicates is represented by error bars. Significant differences between treatments are represented by different letters (*P* < 0.01). SynCom mixture of three *Bacillus* spp. strains.

Compared to the control and *R. solanacearum* treatments, the concentration of JA and SA was significantly increased by SynCom inoculation (Fig. 5D and E). The expression levels of the defense-related marker genes involved in JA and SA signaling in systemic leaves were analysed to identify whether the SynCom activates defense signaling in eggplant. The expression of SA signaling marker genes *EDS1*, *GluA*, *NPR1*, and *SGT1* was upregulated by 2.7-, 6.0-, 3.1-fold, and 1.5-fold, respectively, in eggplant treated with SynCom (Fig. 6A). The expression levels of *TGA*, *PAD4*, and *PR-1a* have no significant difference between *R. solanacearum* and *R. solanacearum* plus SynCom plants (Fig. S11, see online supplementary material). Compared to control and *R. solanacearum* treatment, the expression of JA signaling marker gene *LoxA* upregulated by 12.5-, and 2.1-fold at 48 hpi, respectively, in eggplant treated with SynCom, while the expression of the *Pin2* gene declined compared with the control (Fig. 6B). In addition, SA-biosynthetic gene *ICS1* was increased in *R. solanacearum* plus SynCom treatment plants compared to control and *R. solanacearum*. SA catabolic gene *PBS3* was significantly higher in control than in other treatments (Fig. 6A). These results indicate that the SynCom primed SA- and JA-dependent induced systemic resistance (ISR) against *R. solanacearum* in eggplant.

## Discussion

Host resistance and the rhizosphere microecological balance are the most effective and eco-friendly in controlling BW, as are other soil-borne diseases. Depending on the varieties used, resistance to *R. solanacearum* in eggplant is controlled by one dominant gene, one recessive gene, or recessive polygene [14, 26–28]. Due to the relation of BW resistance with horticultural undesirable traits related to the wild species, the majority of varieties cannot be used directly for breeding. The resistant cultivar R06112 with good quality and commercial property has tremendous potential for introgression into commercial cultivars. *R. solanacearum* resistance in R06112 is controlled by one QTL, located at a 270 kb region, including four *nsLTPs* genes, and the expression levels of two *nsLTPs* genes were significantly upregulated in R06112 by RNA-Seq analysis. *nsLTP* positively mediated the resistance to BW of eggplants by VIGS. nsLTPs belong to the pathogenesis-related protein family, and some of them play a positive regulatory role in plant disease resistance. Study shows that plant resistance to *Phytophthora infestans* was positively regulated by *StLTP10* [29]. *StLTPa7* participates in the early stages of resistance to BW in potatoes [30]. The *NtLTP4*-overexpressing significantly improved the resistance *to R. solanacearum* by increasing the antioxidant enzyme activity, upregulating the expression of defense-related genes, and promoting the stomatal closure of *Nicotiana benthamiana* [31]. Thus, *nsLTPs* were identified as the key candidate genes that may be involved in the resistance of eggplant R06112 to BW. The novel QTL promotes an in-depth understanding of the genetic mechanism of eggplant resistance to BW, and the developed tightly linked KASP markers can be used for new varieties breeding.

The genotype and phenotype of progeny become more similar to the recurrent parent after each backcross generation [32, 33]. Rhizosphere microorganisms have been recognized as the second genome of host plants, which co-evolved with their plants as a meta-organism, and the term ‘holobiont’ has been used to describe the inseparable relationship between them [34]. In this study, the rhizospheric bacterial community of BC_2_F_1_ populations was more similar to S51193 than R06112, which is in agreement with previous reports that rhizosphere bacteria are considered the second genome of the host. In addition, the bacterial α-diversity was higher in EB158 and EB327, which is consistent with the fact that the genotype of hybrid progeny is more complex than that of parents. These results suggested that the differences in bacterial communities in the eggplant rhizosphere are driven by genotypes.

Our study suggests that the abundances of *Bacillus* were significantly higher in the eggplant rhizosphere soil of resistant parent R06112 and the resistant progeny EB158 than in the susceptible progeny EB327 and susceptible parent S51193. Many plant-associated *Bacillus* isolates are reported naturally occurring isolates and can suppress plant pathogens. Numerous studies have been devoted to *Bacillus* spp. they are showing a capacity to control *R. solanacearum*. *Bacillus amyloliquefaciens* PMB05 intensifies plant immune responses to confer resistance against BW of tomato [35]. Cao *et al.* [36] have shown that *B. velezensis* isolates Y6 and F7 have strong antagonistic activity against *R. solanacearum*. Here, *Bacillus* was enriched in suppressive soil including resistant cultivar R06112, and the resistant progeny EB158 (with *EBWR10*), indicating *Bacillus* plays an essential role in *R. solanacearum* inhibition. The QTL greatly recruited the bacterial community in the rhizosphere and this recruitment is inheritable from resistant parent R06112. Yin *et al.* [37] demonstrated that the heritability of tomato rhizobacteria can improve the resistance against BW. Three rhizosphere-associated bacteria were isolated from suppressive soil that showed strong antagonistic activity against *R. solanacearum*.

**Figure 7:**
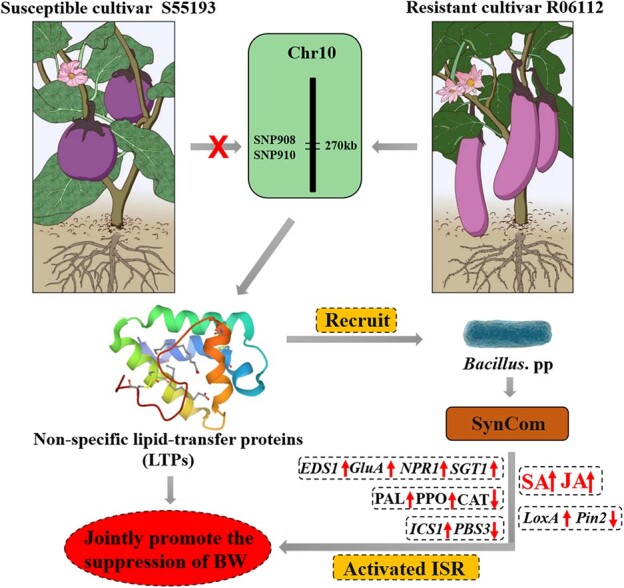
The proposed mechanisms of defense response of eggplant R06112 resistance to *Ralstonia solanacearum*.

Defense enzymes play significant roles in host plant resistance against pathogen invasion. Various signaling molecules, such as O_2_^-^, NO, and H_2_O_2_ are involved in the regulation of multiple signaling pathways and regulate plant defense responses. Phytohormones are vital components of multiple pathways that control plant defense responses by regulating the expression of resistance genes. The alleviations in disease severity in stressed plants have been attributed to changes in the indicators in several species [38]. The disease symptoms were decreased in susceptible cultivar S55193 by SynCom treated. Besides, the PAL, CAT, and PPO activities and the content of O_2_^-^, NO, and H_2_O_2_ were significantly changed by SynCom inoculation. Significantly increased SA and JA levels were observed in *R. solanacearum* plus SynCom treatments. The expression levels of SA and JA signaling marker genes were significantly upregulated. In addition, these bacteria get into the eggplant and tomato plants after their application by identification of copies of *metC* gene in *B. velezensis* PR-1 and *B. velezensis* PR-7, and copies of *cesB* gene in *B.cereus* PR-3.In summary, A synthetic community comprising these three strains displays significant disease resistance against *R. solanacearum* in pot experiments. The SynCom activated SA- and JA-dependent ISR against *R. solanacearum *in eggplant and tomato plants. JA and SA play an important role in systemic resistance and interactions between plants and microorganisms. A previous study showed that the designed SynCom comprising *Brevibacterium frigoritolerans* HRS1, *Bacillus niacini* HRS2, *Solibacillus silvestris* HRS3, and *Bacillus luciferensis* HRS4 can activate JA-dependent ISR against *R. solanacearum* [18]. Similarly, the combination of beneficial rhizosphere bacteria can improve the plants’ ISR and immune response. These results show that introduced native *Bacillus* strains in the rhizosphere of eggplant can be used to control BW under greenhouse conditions. These findings are in agreement with previous reports that the enrichment of rhizosphere-protective microbiota promotes the inhibition of BW [19, 35]. In addition, Kwak *et al.* [19] claimed that BW-resistant tomatoes can recruit beneficial bacterial allies to protect themselves from *R. solanacearum* infection. Present studies reported that plants regulate rhizosphere microbiota to establish disease inhibition [19, 39]. However, how the QTL in disease-resistant varieties affects rhizosphere microbiota is largely unknown. Here, we first demonstrated that the QTL in resistant eggplant greatly modified the bacterial community in the rhizosphere, which jointly confers resistance to *R. solanacearum* (Fig. 7). Our research provides insights into the heritability of *R. solanacearum*-resistant rhizobacteria from resistant parent to progeny is attributed to QTL. In addition, the results indicate a potential for utilizing QTL-driven beneficial microbiota taxa for disease control. The *EBWR10* will be crucial for breeding eggplant with broad-spectrum resistance to BW. Also, the *Bacillus* isolated in our research has good application prospects in the control of BW and promotes plant growth in eggplant and tomato plants. Resistant cultivar breeding and the rhizosphere microecological balance are the most effective and eco-friendly in controlling bacterial wilt. Future investigations are needed to (i) elucidate the function of candidate genes, (ii) identify the fluctuation of bacteria community structure in the rhizosphere with CRISPR/Cas9/sgRNA-mediated targeted gene modification, and (iii) clarify SynCom determinants that prime plant immunity.

## Conclusions

The QTL in R06112 localized at 270 kb region on chromosome 10, namely *EBWR10*, and *nsLTPs* were identified as the key candidate genes controlling BW resistance. In addition, *EBWR10* significantly affects the rhizosphere bacterial community and fosters the recruitment of *Bacillus* in the rhizosphere. The designed SynCom comprising three isolated strains could suppress disease symptoms and activate ISR and plant immune responses against *R. solanacearum* infection. The results of this study indicate the potential for designing microbiota taxa and microbiome-based breeding to improve plant resistance and production. The results also support the theory that the genetic basis of disease resistance assumes the hologenome of the plant and its microbial counterparts are important components of plant defense.

## Materials and methods

### Plant materials

Two inbred eggplant lines, R06112 and S51193, were created and propagated in our lab (Fig. S1A and B, see online supplementary material). The S51193 is highly susceptible to BW (Fig. S1D and E, see online supplementary material), whereas R06112 is highly resistant (Fig. S1F and G, see online supplementary material).

### Bacterial strains

The four *R. solanacearum* strains used in this study—GMI1000, KJ913688, Sm-DgHm-00-2, and Sm-Sg-08-2—belong to phylotype I (Table S1, see online supplementary material). They were chosen according to their degree of aggressiveness on eggplants, and the sequevars 15, 34, and 44 were predominant in Guangdong [40]. All strains are highly aggressive on the susceptible parent S51193.

The strains were cultured in Petri plates containing Kelman’s triphenyl tetrazolium chloride (TTC) agar medium. The inoculated plates were incubated at 30°C for 48 h. Colonies of *R. solanacearum* presenting pink-reddish pigmentation were inoculated in the flasks containing nutrient broth (NB) medium. The flasks were incubated at 30°C for two days. Afterward, bacterial cells were recovered by centrifuging culture media at 4000 × *g* for 10 min. The pellet containing bacterial cells was suspended in autoclaved distilled water. The bacterial population was adjusted to 10^8^ CFU/ml by spectrophotometer.

### Genetic population construction

The non-segregating population (F_1_) was created by crossing S51193 (female parent, Ps) and R06112 (pollen donor, P_r_) (Fig. S1C, see online supplementary material). Similarly, the F_2_ progenies (Fig. S1H, see online supplementary material), BC_1_P_s_, and BC_1_P_r_ were obtained by self-pollinating F_1_ individuals and backcrossing with the parental lines. The controlled hand pollination technique was used to obtain all the crosses in the greenhouse at the Vegetable Research Institute, Guangdong Academy of Agricultural Sciences.

### Inoculation and disease evaluation

The seedlings were carefully uprooted when plants reached at growth stage bearing 4–5 fully expanded leaves. The roots of the plants were dipped in the previously prepared aqueous suspension of *R. solanacearum* for 20 minutes. Afterward, inoculated seedlings were again transplanted in pots containing sterilized artificial potting mix for disease development. The disease index (DI) was calculated according to these criteria: 0 = asymptomatic, no wilting; 1 = minor symptoms with less than 25% wilted leaves; 2 = moderate symptoms with 25–50% wilted leaves; 3 = severe symptoms with 50–75% wilted leaves; 4 = dead plant with 100% wilted leaves, as shown in Fig. S1I–M (see online supplementary material). The disease incidence was calculated using the following formula: wilt rate = the number of plants diseased/the total of plant ×100%. Whereas, the disease severity index (DSI) was calculated using the below-mentioned formula proposed by Du *et al.* [41].

\begin{align*} \text{DSI}=\Sigma\ (\text{disease score}\ \times\ \text{NDP})/\text{N}. \end{align*}

Where NDP is the total number of plants with the specified disease score, N is the total number of plants. The disease response was based on the disease score at 21 days postinoculation (dpi). The disease-related parameters of P_s_, P_r_, F_1_, F_2_, BC_1_P_s_, and BC_1_P_r_, are presented in Table S2. (see online supplementary material)

### Library construction for bulked segregant analysis, sequencing, and QTL analysis

A total of four different libraries including R06112, S51193, R-pool (resistant to BW), and S-pool (susceptible to BW) were used to perform BSA-Seq analysis to identify QTL in R06112 controlling BW resistance. The genomic DNA extracted from 35 resistant (DSI = 0 at 21 dpi) F_2_ individuals and 35 of the most susceptible (DSI = 4 at 7 dpi) F_2_ individuals were mixed in equal quantities to generate R- and S-pools. Afterward, the genomic DNA of both parents, R- and S- pools was sequenced by the Illumina HiSeq platform at Biomarker Technologies Corporation (Beijing, China). The clean sequenced reads were mapped onto the *S. melongena* reference genomes [42]. The aligner Burrows-Wheeler was run with default parameters. SNPs and Insertion/Deletion (InDel) were checked with the GATK algorithm. The filter conditions for obtaining SNP loci according to Luo *et al.* [43]. The ANNOVAR software annotated the gene functions. The significant differences in genotype frequency between the pools shown as Δ (SNP index) were investigated by association analysis based on the SNP index. The candidate regions linked with the disease resistance were confirmed by Δ SNP index exceeding the threshold.

### Development and analysis of KASP markers

The regions linked with the BW resistance as highlighted by the Super-BSA analysis were further verified by performing polymorphic SNPs marker-based QTL analysis. Here we used Primer 5.0 software to develop PCR primers for the competitive allele-specific PCR-single nucleotide polymorphism (KASP-SNP) linked markers. PCR was performed on parental lines to denote the polymorphic efficacy of the designed primers. The polymorphic markers were used on the basis of this preliminary PCR analysis to screen the bulks and backcross population.

### RNA-Seq and virus-induced gene silencing assays of the predicted genes in the QTL region

The roots of S51193 and R06112 treated plants were harvested at 6 h post-inoculation (hpi) and primarily used for RNA-Seq analysis. The RNA samples were sequenced on an Illumina Hi-Seq 2500 platform, and the downstream analyses were performed on the BMK Cloud (www.biocloud.net) at Biomarker Technologies Corporation (Beijing, China). The genes with a fold-change ≥2 and a false discovery rate (FDR) ≤0.05 were identified as DEGs. Virus-induced gene silencing assays were performed using the tobacco rattle virus (TRV) according to You *et al.* [44].

### Rhizosphere soil sampling

The resistant cultivar R06112 (with the QTL), susceptible cultivar S51193 (without the QTL), and the individuals from the BC_2_P_s_ population with the QTL (resistant individuals EB158–94, hereinafter referred to as EB158) or without the QTL (susceptible individuals EB327–77, hereinafter referred to as EB327) were selected by KASP markers, and resistance phenotype (Fig. 1D) were used to analyse the influence of BW-resistant QTL on the rhizosphere bacterial communities. Plants were grown in the field according to a randomized complete block design in Zhongluotan (23°23′N, 113°26′E), Guangzhou City, Guangdong Province, China. Each line consisted of three replicated blocks, with each block measuring 10 m × 8.7 m. The soil type was loam soil. Soil physicochemical conditions were the following: pH, 6.20; organic matter, 3.04%; total N, 0.76 g/kg; total P, 1.2 g/kg; total K, 8.37 g/kg. The same agricultural management practices were used for all the experimental fields. The application of any agrochemicals, such as pesticides, herbicides, or chemical fertilizers was not performed.

Soils from the rhizosphere of the plants were sampled at the time of early fruit formation. For that purpose, the soil adhering to the roots was collected by vigorously shaking. Afterward, a camel hair brush was used to remove the soil from the roots. The soil was collected in sterilized bags. The soil from the roots of ten replicate plants was pooled.

### DNA extraction and sequence analysis of soil samples

MP DNA Isolation Kit (MO BIO Laboratories, Carlsbad, CA, USA) was used for total DNA extraction from 0.5 g soil according to the instructions for use. A universal primer (515F: 5′-GTGCCAGCMGCCGCGGTAA-3′; 806R:5′-GGACTACHVGGGTWTCTAAT-3′) was used to amplify the bacterial 16S rRNA gene. Sequencing libraries were constructed by TruSeq® DNA PCR-Free Sample Preparation Kit (Illumina) by the instructions. After quality evaluation, the libraries were deep sequenced at Novogene Co., Ltd (Beijing, China) on an Illumina NovaSeq platform, and 250 bp paired-end reads were generated.

The high-quality 16S rRNA sequences were filtered and used for analyses. Standalone BLASTN analysis against a SILVA 16S rRNA gene database was conducted on sequences that removed the barcode regions and primer along with chimeric sequences by ChimeraSlayer software. Sequences with ≥97% similarity were designated to the same OTUs. The multiple sequence alignment was used to investigate the phylogenetic relationship of different OTUs and the difference of the dominant species between samples performed by the MUSCLE software (Version 3.8.31, http://www.drive5.com/muscle/). QIIME (Version 1.7.0) was used to calculate Alpha diversity (α-diversity), including Shannon and Chao1. R software (Version 2.15.3) was used to show α-diversity. QIIME software (Version 1.9.1) was used to calculate the Beta diversity (β-diversity) of the rhizospheric samples. The WGCNA package, stat packages, and ggplot2 package in R software (Version 2.15.3) were used for Principal Coordinate Analysis (PCoA) analysis. QIIME software (Version 1.9.1) was used for the Unweighted Pair-group Method with Arithmetic Means (UPGMA) Clustering analysis.

### Isolation and evaluation of antagonistic bacteria

The biocontrol strains were isolated from the rhizosphere soils of EB158 individuals. Soil samples were homogenized into sterile distilled water at 150 rpm for 15 min. Soil suspensions with serial dilutions concentrations were spread on Luria broth (LB) medium and incubated at 30°C for 24 h, and suspected colonies of bacteria were selected.

### The antagonistic ability of isolates against *R. solanacearum in vitro*

The purified clones have been identified by antagonistic activity against *R. solanacearum* GMI1000 on the plate to identify antagonistic response by dual culture method [45]. A total of 10 μL suspension of each isolate (OD600 = 1.0) was dripped on the *R. solanacearum*-inoculated plates, and the plates were incubated at 30°C, and the inhibition zone was investigated after 48 hours.

### Identification of the isolated strains

The identification of antagonistic strains against *R. solanacearum* was performed by 16S rRNA gene sequence and Average Nucleotide ldentity (ANI) using BLAST and aligned against sequences of reference strains in the NCBI GenBank database. Molecular Evolutionary Genetics Analysis (MEGA) software version 5.0 was used to construct phylogenetic trees by the maximum likelihood method.

### Evaluation of BW suppression by the synthetic consortium

To conduct the SynCom (composed of isolated strains) treatment in a pot experiment, the final OD600 of mixed suspension culture was adjusted to 1.0. The susceptible S51193 plants were infected with *R. solanacearum* (a mixture of four *R. solanacearum* strains described in Table S1 (see online supplementary material), the bacterial population was adjusted to 10^8^ CFU/ml) by irrigating roots, as mentioned above. The plants were collected at 48 hpi, and the roots were cleaned with sterile water. Secondary messengers (NO, O_2_^−^, H_2_O_2_) and Defense-related enzyme (CAT, PPO, PAL) activity were measured by commercial chemical assay kits (Nanjing Jiancheng Bioengineering Institute, Nanjing, China). Phytohormone levels were measured according to Gong *et al.* [38]. The gene-specific primers used for real-time reverse transcription-PCR (qRT-PCR) to investigate defense signaling marker genes in eggplant are listed in Table S3–S4(see online supplementary material). Triplicate qRT-PCR reactions were conducted for samples. The 2^−ΔΔ^Ct method was used for the analysis of the relative gene expression data [46].

### Statistical analysis

Genome Sequence Archive [47] in the National Genomics Data Center, China National Center for Bioinformation/Beijing Institute of Genomics, Chinese Academy of Sciences was used to deposit the raw sequence data (GSA: CRA008448, CRA009725) [48], which are publicly accessible at https://ngdc.cncb.ac.cn/gsa. All data were presented as mean ± standard deviation (SD). One-way analysis of variance (ANOVA) in SPSS software (SPSS, Chicago, IL, USA) was used for statistical analysis. Separations were conducted by Duncan’s multiple-range tests. Differences at *P* < 0.01 were regarded as statistically significant.

## Acknowledgements

The present study was financially supported by major special projects of the Guangxi Science and Technology Program (AA22068088-2), the Guangdong Basic and Applied Basic Research Foundation (2021A1515012490), the Natural Science Foundation of China (32372702), the Guangzhou Basic and Applied Basic Research Foundation (202201010247), the Department of Agriculture and Rural Areas of Guangdong Province of China (2023KJ110, 2023KJ106, 2022-NJS-00-005, and 2022-NPY-00-026), the Special Fund for Scientific Innovation Strategy-Construction of High Leveled Academy of Agriculture Science (202114TD, R2019PY-JX003, R2023PY-JX008) and the Scientific and technological research project of Harbin Science and Technology Department (2021ZSZZNS07).

## Authors’ contributions

C.G., T.L. and Z.W. contributed to the idea, performed the experiments, analysed and visualized the data, and wrote the original manuscript. S.C. and P.M. performed the experiments. W.L. and S.L. made contributions to the data analyses. Z.L., B.S., Z.L., and Y.L. provided the resources and supervised the project. T.L. and Y.W. were major contributors to conceptualization, project administration, supervision, and funding acquisition.

## Data availability

Genome Sequence Archive in the National Genomics Data Center, China National Center for Bioinformation/Beijing Institute of Genomics, Chinese Academy of Sciences was used to deposit the raw sequence data (GSA: CRA008448, CRA009725), at https://ngdc.cncb.ac.cn/gsa. All relevant data generated or analysed are included in the manuscript and the supporting materials.

## Conflict of interest statement

The authors declare that there are no conflicts of interest.

## Supplementary data

Supplementary data is available at *Horticulture Research* online.

## Supplementary Material

Web_Material_uhad272
