# Supplementary material for: A QTL of eggplant shapes the rhizosphere bacterial community, co-responsible for resistance to bacterial wilt
Source: Hortic Res. 2023 Dec 19;11(2):uhad272. doi: 10.1093/hr/uhad272 (PMC10852381; doi:10.1093/hr/uhad272)
Supplement: Web_Material_uhad272 [file web_material_uhad272.docx]

**A QTL of eggplant shapes the rhizosphere bacterial community, co-responsible for resistance to bacterial wilt**

Chao Gong ^a,1^, Zhenshuo Wang ^b,1^, Zhiliang Li ^a^, Baojuan Sun ^a^, Wenlong Luo ^a^, Shanwei Luo ^a^, Shuting Chen ^a^, Peiting Mai ^a^, Zhenxing Li ^a^, Ye Li ^c^, Yikui Wang ^d*^, Tao Li ^a*^

**Supplementary File**


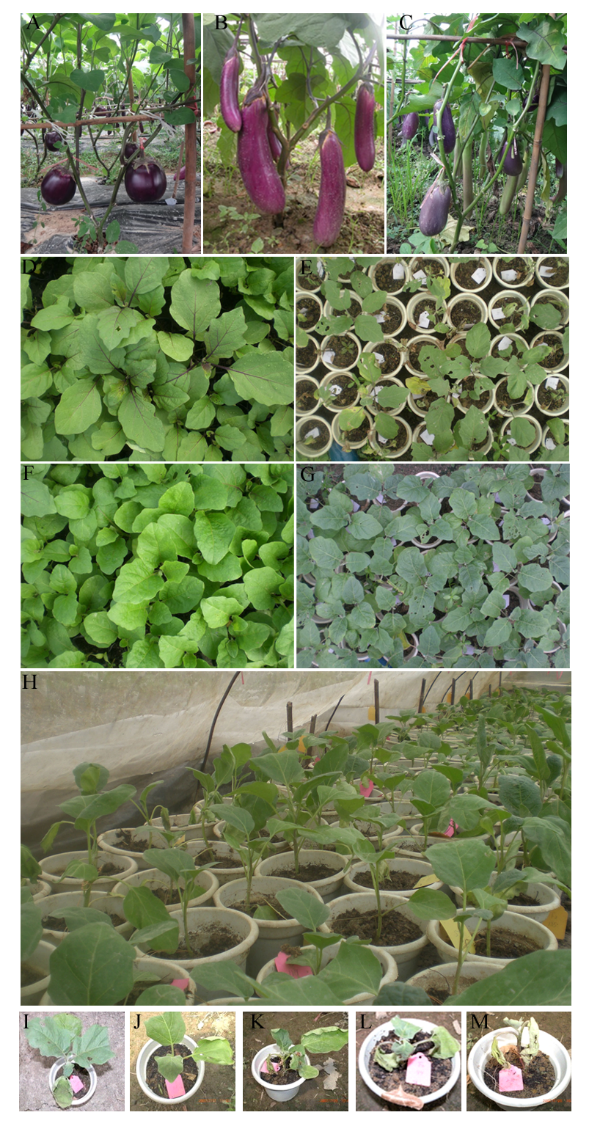


Figure S1. The characteristics of bacterial wilt (BW) susceptible parent S51193 and resistant parent R06112, and the phenotype of 21 days after inoculation with *R. solanacearum*. The characteristics of susceptible eggplant cultivar S51193 and BW-resistant eggplant cultivar R06112 at fruit maturation period have been shown in (A) and (B), respectively; (C) the phenotype of F1 generation; (D) and (E) the characteristics of S51193 and disease symptoms on S51193; (F) and (G) the characteristics of R06112 and disease symptoms on R06112 at 20 days post-*R. solanacearum* inoculation (dpi). (H), the phenotype of F2 generation; (I)-(M), disease severity of BW in eggplant. “I”, “J”, “K”, “L”, and “M” represent 1, asymptomatic (no wilting), 2, minor wilting (25%), 3, moderate wilting (50%), 4, severe wilting (75%) and 5, plant death (100%), respectively.


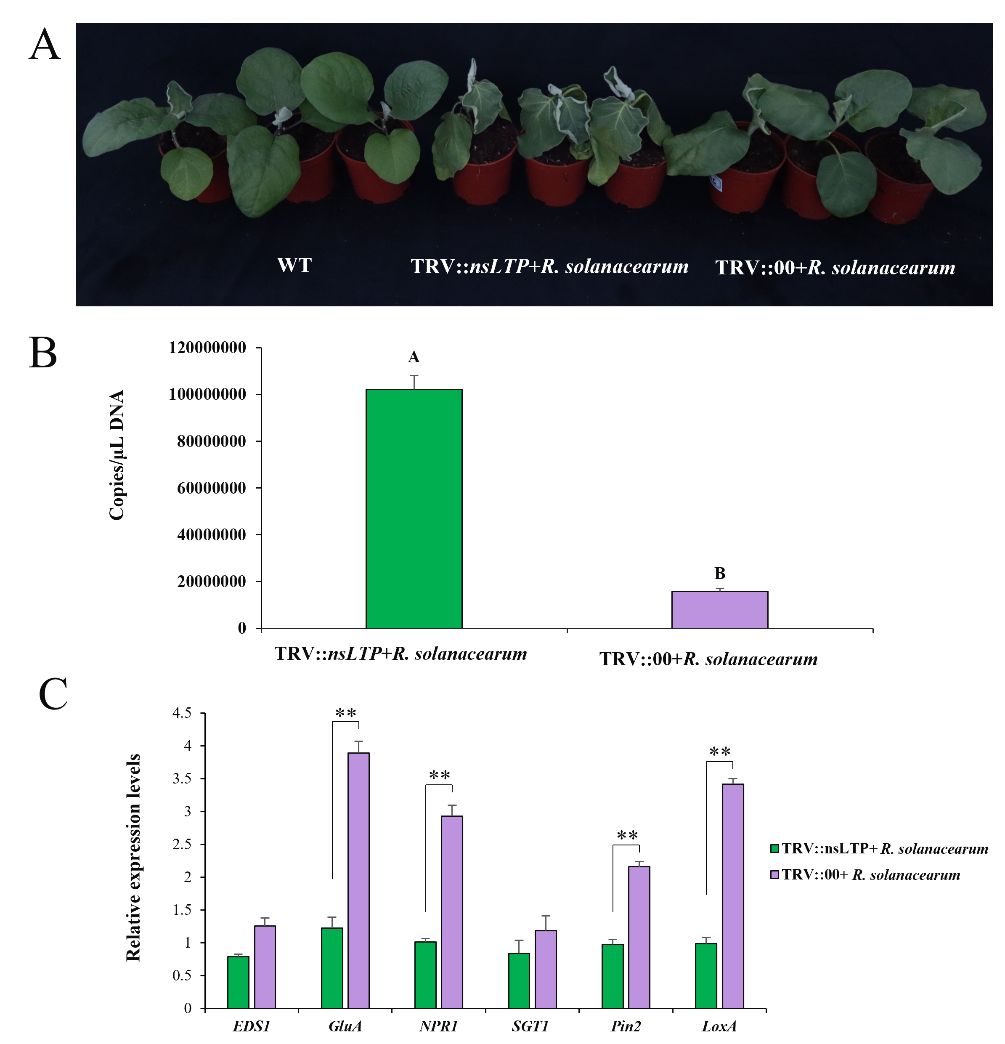


Figure S2. Silencing *nsLTP* reduced eggplant resistance to bacterial wilt by VIGS analysis. *nsLTP* (SMEL_010g351470.1) expression in the resistant cultivar R06112 was silenced by VIGS. (A) Phenotypes of the control and *nsLTP* silenced lines inoculation with *R. solanacearum* at 7 dpi; (B) Copies of the *Deacetylase* gene in *R. solanacearum*; (C) The expression levels of pathogen-induced genes associated with SA and JA response in control and *nsLTP* silenced plants infected with *R. solanacearum* by qRT-PCR analysis. The standard deviation for three independent replicates is represented by error bars. Significant differences between treatments are represented by ** (P < 0.01).


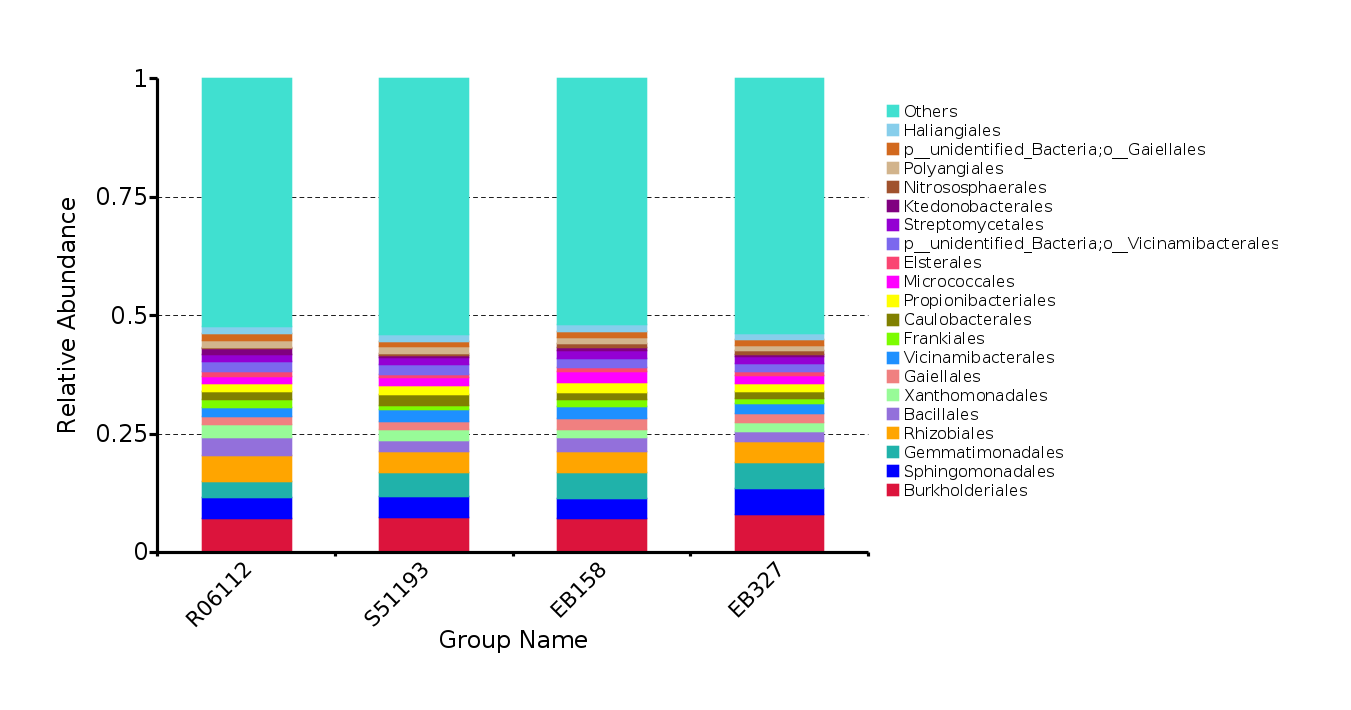


Figure S3. Relative abundance of the most dominant bacterial orders in eggplant rhizosphere.


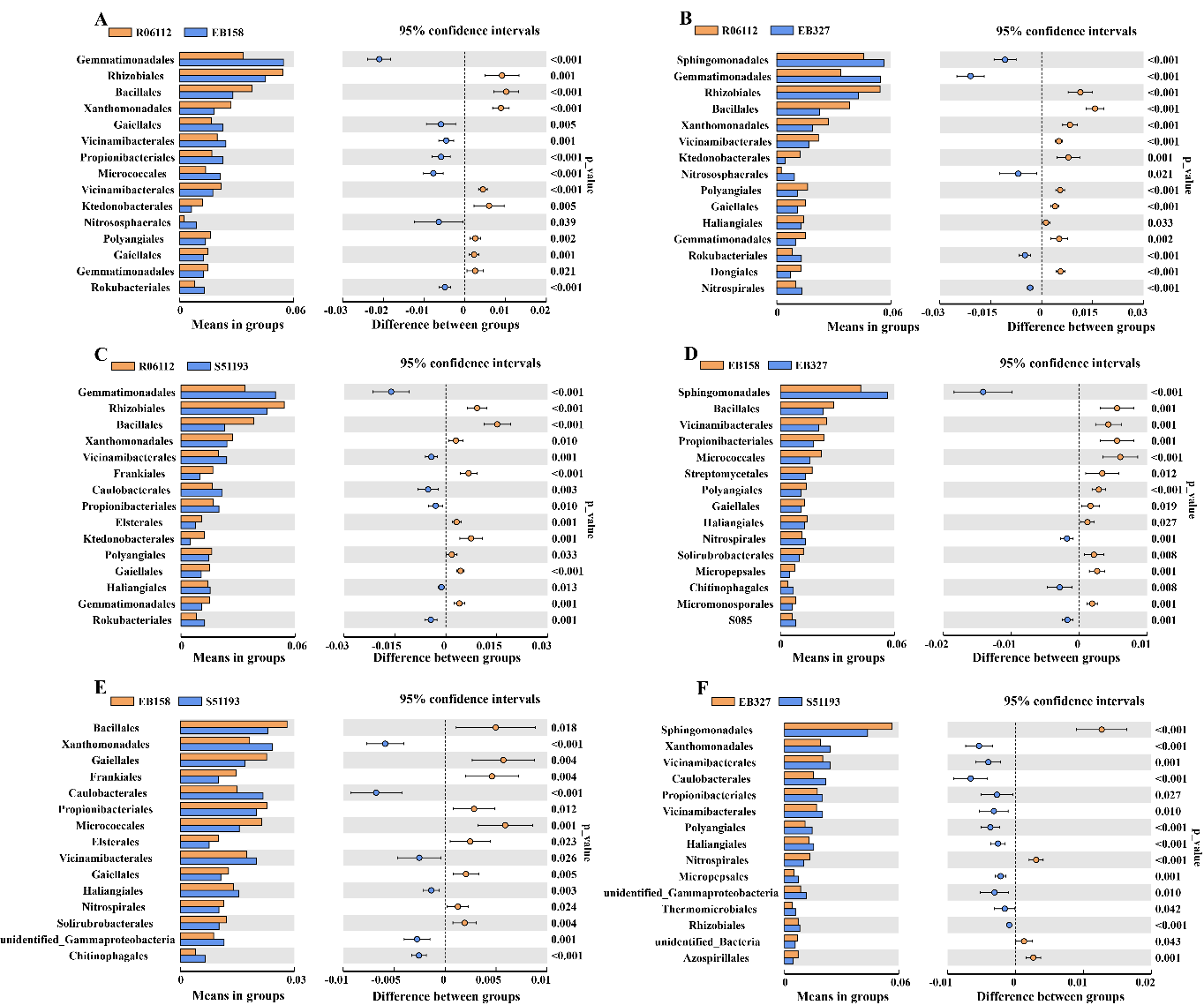


Figure S4. Bacterial orders with significantly different levels of relative abundance. Taxonomic profile of bacterial orders whose relative abundance was different between R06112 and EB158 (A), R06112 and EB327 (B), R06112 and S51193 (C), EB158 and EB327 (D), EB158 and S51193 (E), EB327 and S51193 (F) plots.


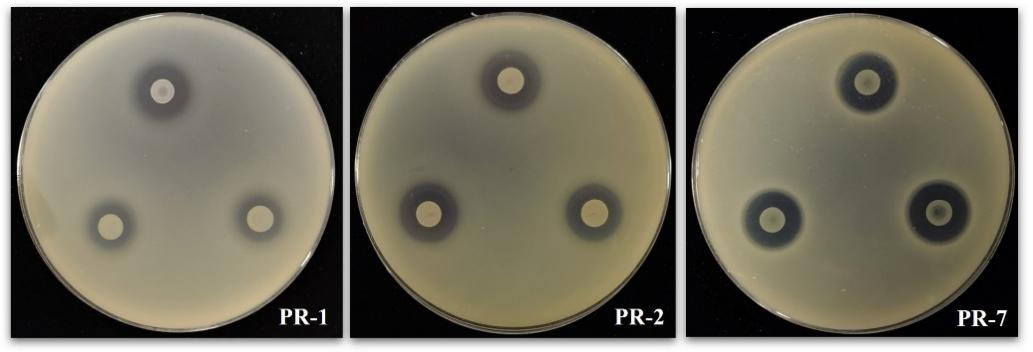


Figure S5. Antagonistic activity of strains *Bacillus velezensis* PR-1, *Bacillus cereus* PR-3 and *Bacillus velezensis* PR-7 against *R. solanacearum*.


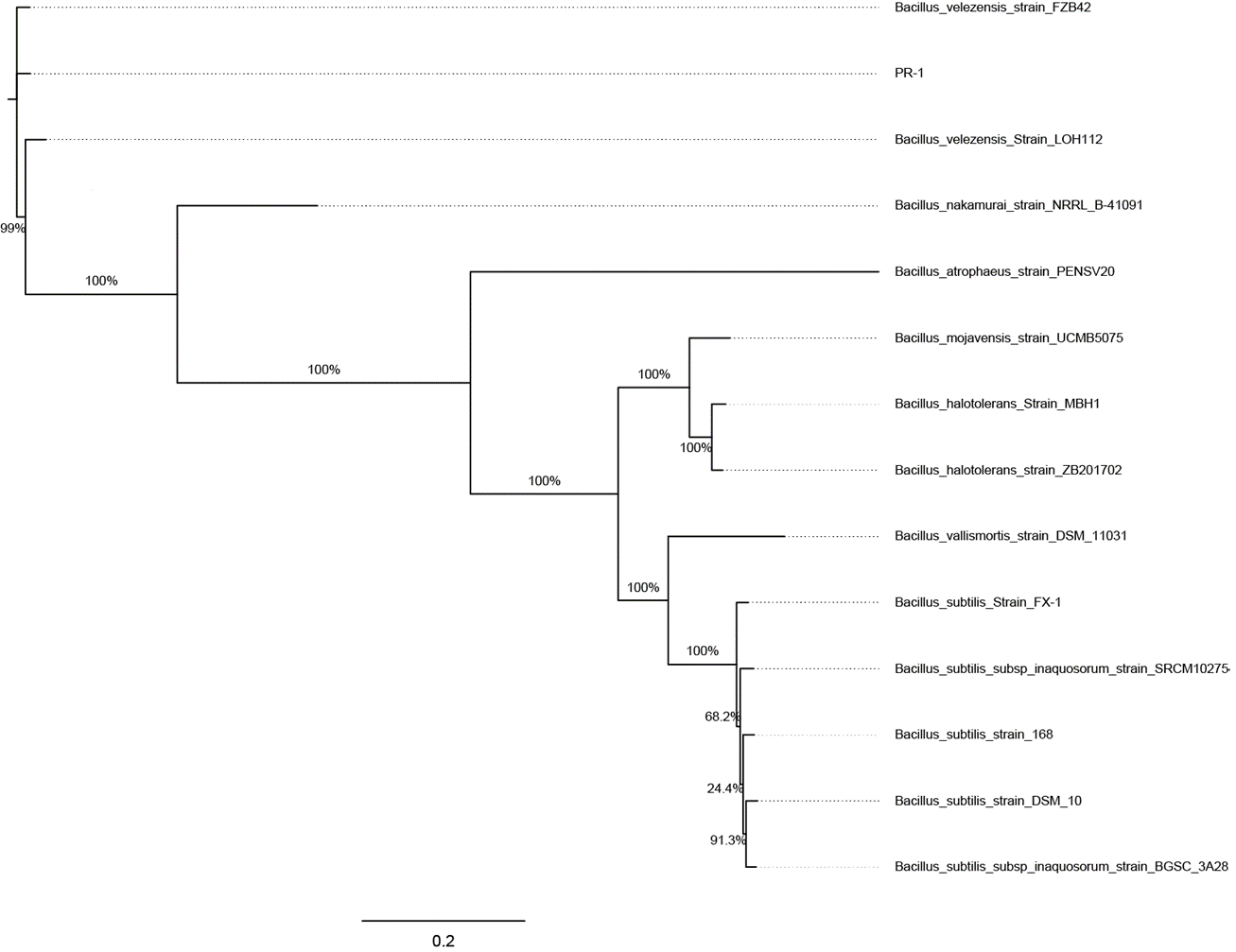


Figure S6. Phylogenetic analysis of strain *Bacillus velezensis* PR-1. 16S rRNA gene sequence and Average Nucleotide ldentity (ANI) using BLAST and aligned against sequences of reference strains in the NCBI GenBank database. Phylogenetic trees were inferred using the maximum likelihood method from Molecular Evolutionary Genetics Analysis (MEGA) software version 5.0.


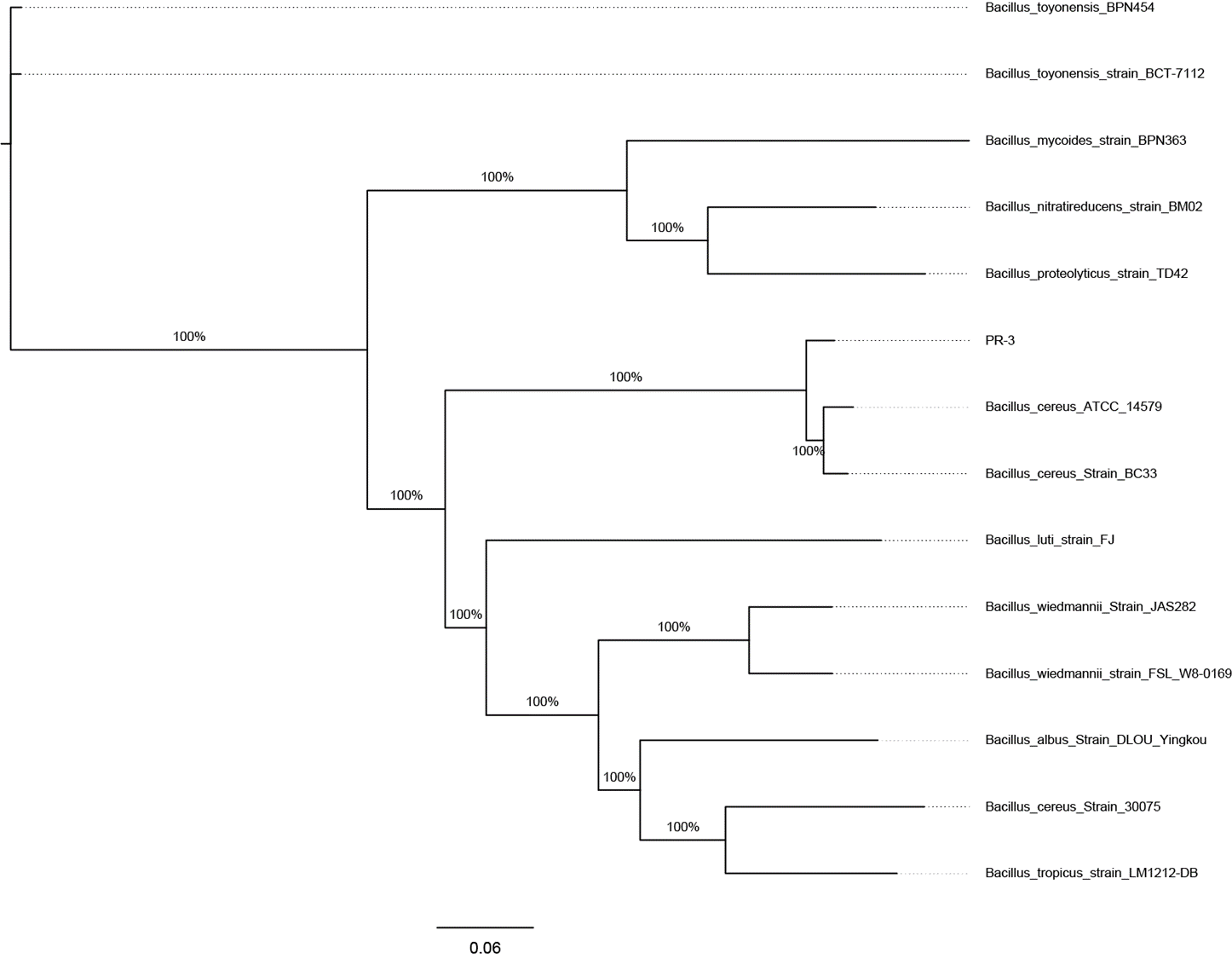


Figure S7. Phylogenetic analysis of strain *Bacillus cereus* PR-3. 16S rRNA gene sequence and Average Nucleotide ldentity (ANI) using BLAST and aligned against sequences of reference strains in the NCBI GenBank database. Phylogenetic trees were inferred using the maximum likelihood method from Molecular Evolutionary Genetics Analysis (MEGA) software version 5.0.


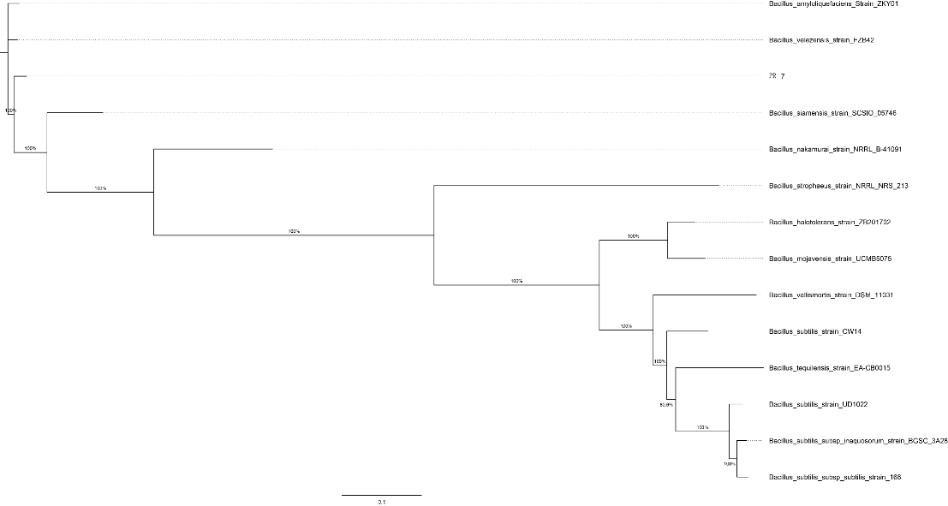


Figure S8. Phylogenetic analysis of strain *Bacillus velezensis* PR-7. 16S rRNA gene sequence and Average Nucleotide ldentity (ANI) using BLAST and aligned against sequences of reference strains in the NCBI GenBank database. Phylogenetic trees were inferred using the maximum likelihood method from Molecular Evolutionary Genetics Analysis (MEGA) software version 5.0.


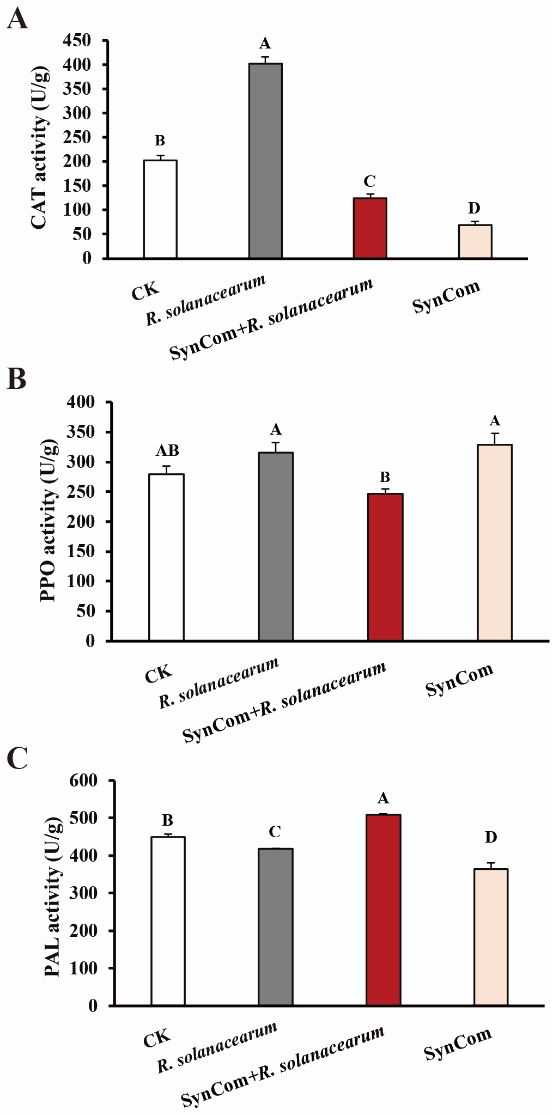


Figure S9. Effects of SynCom on the activities of CAT (A), PPO (B), and PAL (C) of eggplant plants infected with *R. solanacearum* at 48 hpi. Error bars represent the standard deviation for three independent replicates. Different letters indicate significant differences between treatments (P < 0.01). SynCom mixture of three *Bacillus* spp. strains.


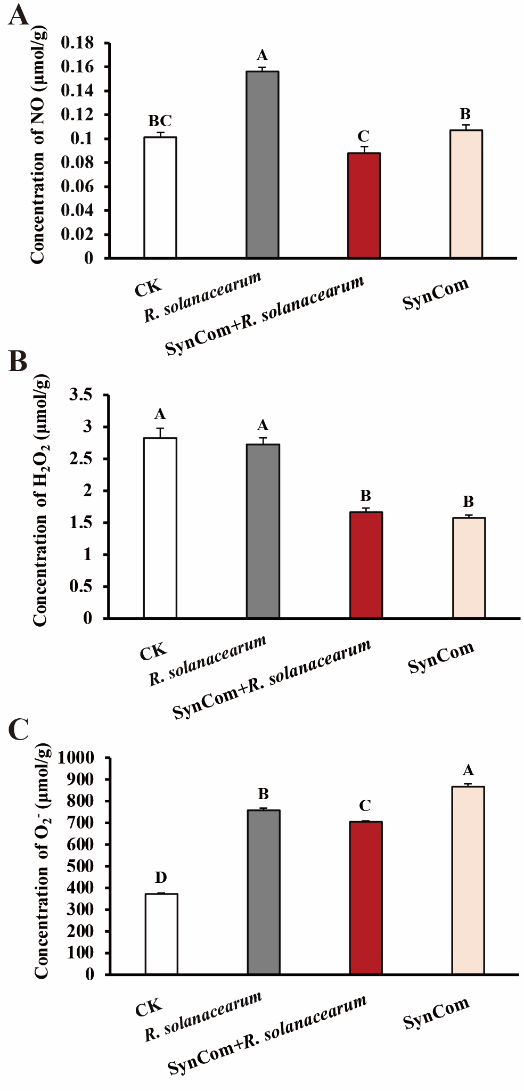


Figure S10. Effects of SynCom on the contents of NO, H_2_O_2_, and O_2_^-^ of eggplant plants infected with *R. solanacearum* at 48 hpi. Error bars represent the standard deviation for three independent replicates. Different letters indicate significant differences between treatments (P < 0.01). SynCom mixture of three *Bacillus* spp. strains.


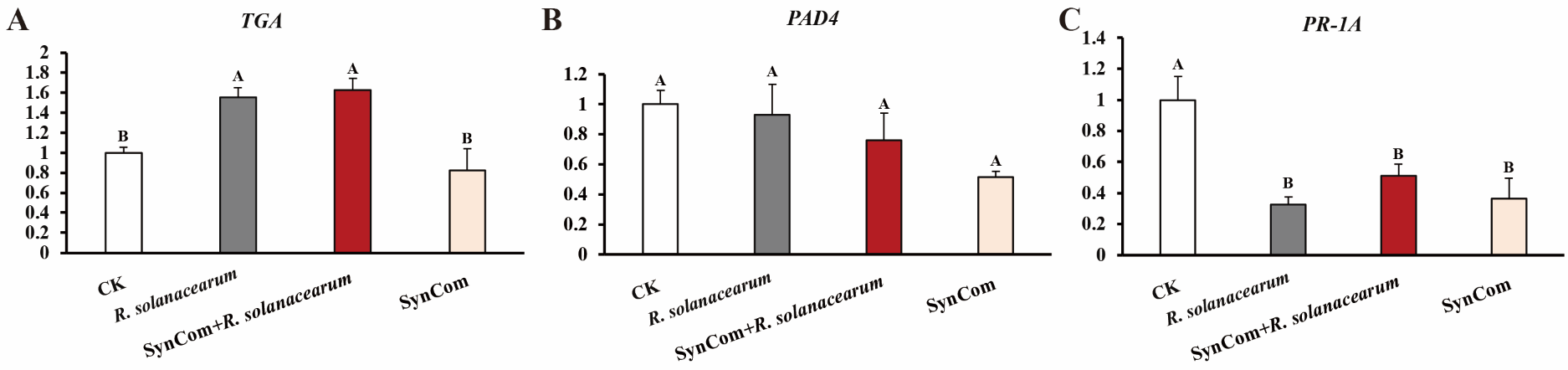


Figure S11. qRT-PCR analysis of salicylic acid (SA) signaling marker gene expression in eggplant plants treated with the SynCom or *R. solanacearum* plus SynCom at 48 hpi with *R. solanacearum*. Error bars represent the standard deviation for three independent replicates. Different letters indicate significant differences between treatments (P < 0.01). SynCom mixture of three *Bacillus* spp. strains.

Table S1. Characteristics of *R. solanacearum* strains used in the study

| Strain | Host of isolation | Geographical origin | Phylotype–sequevar |
| --- | --- | --- | --- |
| GMI1000 | Tomato | French | I-18 |
| KJ913688 | Tomato | Guangdong, China | I-34 |
| Sm-DgHm-00-2 | Eggplant | Guangdong, China | I-15 |
| Sm-Sg-08-2 | Eggplant | Guangdong, China | I-44 |

Table S2. Genetic analysis of bacterial wilt resistance.

| Populations | Pedigree | Segregation | | | R: S  ratio | χ2 | P value in χ2 text |
| --- | --- | --- | --- | --- | --- | --- | --- |
|  |  | Plants tested | Resistance plants | Susceptible plants |  |  |  |
| P_r_ | R06112 | 178 | 174 | 4 | 1:0 | - | - |
| P_s_ | S51193 | 185 | 0 | 185 | 0:1 | - | - |
| F_1_ | R06112🞨S51193 | 123 | 123 | 0 | 1:0 | - | - |
| BC_1_P_r_ | (R06112🞨S51193) 🞨R06112 | 128 | 128 | 0 | 1:0 | - | - |
| BC_1_P_s_ | (R06112🞨S51193) 🞨S51193 | 128 | 66 | 62 | 1:1 | 0.13 | 0.72 |
| F_2_ | R06112🞨S51193 | 320 | 226 | 94 | 3:1 | 3.27 | 0.07 |

^a^ Ps and Pr are the susceptible parent line S5519 and resistant parent line R061121, respectively. The statistics of the phenotypic data was counted during 21 dpi.

Table S3. Bacterial α-diversity index for rhizosphere soil of R06112, S51193, EB158 and EB327.

|  | R06112 | S51193 | EB158 | EB327 |
| --- | --- | --- | --- | --- |
| Chao1 | 3219.89±40.54b | 3223.66±17.50b | 3413.07±22.12a | 3306.7±30.33ab |
| Shannon | 10.06±0.014bc | 10.04±0.017c | 10.16±0.009a | 10.1±0.014b |

The significance of the differences between R06112, S51193, EB158, and EB327 was significantly shown by different letters (p ＜ 0.05).

Table S4. The primer sequence of qRT-PCR

| Gene name | Forward(5’-3’) | Reverse(5’-3’) | Gen Bank accession |
| --- | --- | --- | --- |
| *Pin2* | TGATGCCAAGGCTTGTACTAGAGA | AGCGGACTTCCTTCTGAACGT | AY129402 |
| *LoxA* | GACCAAAACGCTCGTCTCTC | TGGTAGACCACCAACACGAA | U09026 |
| *EDS1* | GTTTCGCAGACAAGTTGAGCC | CTCTGTGTGAACCGATAACGC | AY679160.1 |
| *GluA* | GCCGACTGGGTGAGATGGTAA | ACATTGTTGTGCCCGTGGAC | M80604 |
| *NPR1* | CTTGGACTGGGTGTTGCTAATG | TGCCCATCCAATGTAATGTCTG | NM_001247633.1 |
| *TGA* | GCAAGTGACCCTGAACTACGAAG | GGGTTTTCCACATCCCTGACAAG | GQ386946.1 |
| *SGT1* | TTCTCGGTTTTGAGGAAGGG | GCAGATACCAAGTGATGTCTACCA | NM_001247758.1 |
| *PAD4* | ACATCGGCTGAAACCTCCTTATT | TTTGATAAGTGGTGGGGAAATGA | AY753546.1 |
| *PR-1a* | GAGGGCAGCCGTGCAA | CACATTTTTCCACCAACACATTG | M69247 |
| *ICS1* | GCATGGGACAATGCTGCTGCCTCATGGA | TCTGGTGCTACGAGCAAGTACCACCT | NM_001247865.1 |
| *PBS3* | GTTTGCACTATGTATGCTTCCTCTG | GCTCATACTCCTGACCAATCTTTGTC GACA | XM_004251437.2 |
